# Mindfulness to improve the mental health of university students. A systematic review and meta-analysis

**DOI:** 10.3389/fpubh.2023.1284632

**Published:** 2023-12-04

**Authors:** Ana María González-Martín, Agustín Aibar-Almazán, Yulieth Rivas-Campo, Yolanda Castellote-Caballero, María del Carmen Carcelén-Fraile

**Affiliations:** ^1^Department of Education and Psychology, Faculty of Social Sciences, University of Atlántico Medio, Las Palmas de Gran Canaria, Spain; ^2^Department of Psychology, Higher Education Center for Teaching and Educational Research, Madrid, Spain; ^3^Department of Health Sciences, Faculty of Health Sciences, University of Jaén, Jaén, Spain; ^4^Faculty of Human and Social Sciences, University of San Buenaventura-Cali, Santiago de Cali, Colombia

**Keywords:** mindfulness, mental health, university students, systematic review, meta-analysis

## Abstract

**Objectives:**

This systematic review and meta-analysis was to analyze the effects of a mindfulness program on mental health in university students.

**Methods:**

A systematic review was conducted using the databases Pubmed, Scopus, Web of Science and CINAHL. The selected studies had to incorporate a mindfulness intervention in university students. The methodological quality of the collected articles was evaluated using the PEDro scale.

**Results:**

We initially identified 321 studies, of which 21 were included in this review. The interventions focused on mindfulness with durations ranging from 8 weeks to 3 months. The results demonstrated significant effects of a mindfulness intervention on mental health in university students.

**Conclusion:**

This systematic review and meta-analysis found that mindfulness is effective for improving the mental health of college students.

**Systematic Review Registration:**

identifier: CRD42023441453.

## 1 Introduction

The main objective of university education is to reinforce the intellectual abilities of students, as well as to prepare them for productive and successful lives as adults (1). However, in this new stage of life, students face new challenges, such as starting an in-dependent life, meeting new people and, for the majority, moving away from home and their families for the first time (2). This can significantly affect students' wellbeing and mental health leading to, among other issues, considerable worry, sleep problems, stress-related difficulties and high levels of psychological distress. These issues affect the academic success of students (3).

Recent studies have identified several factors that can influence the level of psychological distress in students such as academic difficulties and poor performance, desire for success, adaptation to the university environment, and concerns about their future (2, 4, 5). These difficulties begin in college and, although they can be reduced with mindfulness training, they often do not return to pre-college levels (6). Since the risk of mental disorders increases in early adulthood, college students are a vulnerable population (7). In recent years, there has been increasing interest in the wellbeing of college students and their mental health, driven by a significant increase in mental health problems in this population (8). According to a report, carried out in 2023, by the Ministries of Universities and Health, 52.3% of university students have consulted with health professional for a mental health issue. From them, 46.90% presented depressive symptoms and 52.80% moderate to severe anxiety (9). These psychological problems not only have significant consequences for students, such as increased stress, decreased academic performance, and deterioration in quality of life, but also for university institutions (10). To address the problems facing university students, it is necessary that the university education system encourages the development of socio-emotional competences well as academic skills (11). However, despite growing concern, existing research on college student mental health has overlooked several crucial areas (12). First, there is a lack of longitudinal studies that follow students throughout their college journey to understand how their mental health needs and challenges evolve (13). Furthermore, most studies focus on the identification of mental health problems rather than examining in depth effective strategies and approaches to address these issues (14). In particular, there is an evident paucity of research focusing on interventions aimed at promoting healthy coping skills in this population (15). Approaches have often focused on identifying risk factors, without providing concrete solutions to strengthen students' resilience and coping skills (16). This gap in the literature is significant, as coping skills are critical to psychological wellbeing and successful adaptation to the demands of college life (17). Given the substantial mental health implications of college students, it is imperative to address these gaps in research and practical approaches to promoting psychological wellbeing in the college environment. It is the position of this paper that psychological wellbeing is more than the absence of psychological distress (18). To better understand the college experience, it is essential to address both the challenges students face and aspects of positive functioning. As we explore the challenges, the need arises for interventions that not only alleviate distress but also improve students' coping skills (15). In this context, an area of interest in psychological research is the action-emotion style, known as achievement motivation, and its influence on the way students cope with stress and their academic performance. It is also crucial to consider how these coping skills can be strengthened (19). This interest in the wellbeing of college students has led to the exploration of innovative approaches, such as mindfulness, in the educational environment (20).

Mindfulness is a meditation technique that focuses on the present and encourages acceptance of emotions as they are, without trying to control them. Examples of established practices include Mindfulness-Based Cognitive Therapy (MBCT), Mindfulness-Based Stress Reduction (MBSR), Acceptance and Commitment Therapy (ACT), and Dialectical Behavior Therapy (DBT). These therapies have been shown to be effective in reducing stress, improving mental health, and promoting wellbeing in a variety of contexts, including education (21). Its application has expanded to non-medical contexts, such as education, where its value is increasingly recognized (22). However, there has been little research into whether mindfulness can influence perceptions of the educational environment. For example, a study of more than 600 students in the United Kingdom found that 8-week mindfulness courses could prevent mental illness and improve student wellbeing (23). Additionally, evaluation of university mindfulness programs has shown improvements in students' resilience, self-awareness, and concentration, which benefits their learning experience and work management (24). These results highlight the importance of developing effective motivational programs to foster self-acceptance and personal growth in higher education (25). The promising results suggest that educational institutions should consider integrating mindfulness to improve students' emotional wellbeing, learning potential, and health (26). This is crucial, as student mental health is a topic of growing interest. This is important since student mental health is an issue of increased inter-national concern as it directly influences the development of future professionals and citizens (27). Therefore, the objective of this study is to analyze the effects of a mindful-ness-based program on mental health in university students. Based on this general objective, a series of hypotheses were formulated: (i) Participation in a mindfulness-based program is expected to significantly improve the mental health of university students, reducing levels of stress, anxiety, and symptoms of depression compared to with a control group that does not participate in the program; (ii) The positive effects of the mindfulness-based program on the mental health of university students are expected to last over time, demonstrating sustained improvements in psychological wellbeing and coping ability even after completion of the program.

## 2 Materials and methods

The present research is a systematic review with meta-analysis that seeks to identify the effects of mindfulness as a therapeutic intervention for mental health in university students both in and out of the classroom. This study followed the methodological guidelines as outlined in “An updated guideline for reporting systematic reviews,” a version of the PRISMA document published in the British Medical Journal (BMJ Clin Res Ed) in 2021 (28) and the Cochrane manual for the elaboration of Systematic Reviews of Interventions (29) and its protocol was registered in PROSPERO under the code CRD42023441453.

### 2.1 Sources of information

A comprehensive literature review was conducted in June and July 2023, encompassing databases such as Pubmed, Scopus, Web of Science, and CINAHL. Researchers systematically collected the most current available literature during the period from June 21 to July 18, 2023.

### 2.2 Search strategy

The search terms and boolean operators used to combine them are as follows: Search terms: (“Mind-Body” OR “Mindfulness therapy” OR “MBI” OR “Mindfulness-based intervention”) AND (“university students”) AND (“Depression” OR “Anxiety” OR “psychological distress” OR “mental health” OR “Wellness”).

These terms were combined using boolean operators (OR and AND) to ensure the retrieval of relevant literature addressing the relationship between mindfulness-based interventions and the mental health of university study. Filters were applied in the database to ensure the inclusion of only relevant documents. These filters included: Document Type: The search was limited to documents of the type “Clinical Trial” and “Randomized Control Trial” to ensure the selection of relevant clinical research.

### 2.3 Inclusion criteria

Articles had to meet the following inclusion criteria: (i) the study was a clinical trial or randomized control trial; (ii) the mindfulness intervention was used in undergraduate university students; (iii) objective measures of mental health (depression, anxiety, stress, wellbeing) were taken before and after exercise intervention.

### 2.4 Exclusion criteria

The study excluded research that did not assess relevant variables or primarily focused on populations outside the intended scope, such as ethnic minorities, individuals with limited mobility, those with acute infections, neurological diseases, hormonal disorders, and individuals with a history of psychiatric disorders. These exclusions were made to assess the efficacy of mindfulness-based interventions among university students within a general context. Measures were taken to reduce potential variations arising from non-target populations.

Additionally, studies that did not meet acceptable levels of internal and external validity were excluded. The research also omitted publications such as books, papers, meta-analyses, systematic reviews, protocols, clinical trial registries, and articles that had not undergone peer-review.

### 2.5 Study selection process

The search results were subjected to a processing process using the Rayyan QCRI (https://rayyan.qcri.org/welcome) tool, where automatic procedures were carried out to discard duplicates. Two of the authors read the titles and abstracts, blindly and independently verifying compliance with the inclusion criteria, and subsequently read the articles in their entirety. In cases where discrepancies were identified, a third author, who was an expert in the subject matter, was given access to the list of articles in Rayyan and had the opportunity to independently classify the articles for inclusion. This third decision was used to settle any differences.

### 2.6 Data extraction

The primary variables were mental health outcomes. They were categorized according to several criteria: the type of variable evaluated (such as depression, anxiety, psychological distress, stress); the year of publication; country; author(s); participant characteristics (including age, sample size, and distribution of groups); the intervention (covering duration, intensity and frequency); the scale used for each variable; measurement and follow-up time; and finally, the related statistical data.

### 2.7 Assessment of methodological quality

The methodological quality of the articles included in this review was assessed using the PEDro scale. PEDro scores were obtained from the official website when available, otherwise, articles were evaluated by two authors independently. The PEDro scale consists of eleven items. The first item exclusively addresses external validity and is not added to the final score. Items two through eleven are evaluated based on their presence (one) or absence (zero) within the publication, and they are assigned a score ranging from zero to one. This means the publication's total score can range from zero (minimum) to ten points (maximum). Articles receiving less than four points are categorized as “deficient,” those scoring four to five points are considered “fair,” while those with scores between six and eight are classified as “good,” and publications achieving a score between nine and ten are labeled as “excellent.”

The information was provided regarding the population size of each group, the type of intervention implemented in both the experimental and control groups, the duration of the intervention, the variables under consideration, and the assessment tools employed. For the variable “mindfulness,” findings were reported using the Mindful Attention Awareness Scale and the Five Facets Mindfulness Questionnaire (FFMQ). For the “stress” variable, data was recorded using the Perceived Stress Scale or the Chronic Stress Screening Scale. In the case of “depression,” assessments were conducted using the Self-Rating Depression Scale, Beck Depression Inventory, Depression, Anxiety, and Stress Scale-21, ACS-depression (Affective Control Scale), and the Patient Health Questionnaire-9. For “anxiety,” results were processed based on the ACS-anxiety, Self-rating Anxiety Scale (SAS), Beck Anxiety Inventory, and Trait Anxiety Inventory. Additionally, for each study, the measurement stages and outcomes were reported.

Within the realm of our research, we harnessed a diverse array of tools to scrutinize the multifaceted aspects of mindfulness, stress, depression, and anxiety, all playing integral roles in the mental wellbeing of university students. The Mindful Attention Awareness Scale and the Five Facets Mindfulness Questionnaire (FFMQ) emerged as stalwart instruments. These instruments, like attentive sentinels, delved deep into the intricate dimensions of mindfulness, and from awareness to acceptance. They provided us with a panoramic view of an individual's mindfulness quotient, unveiling its potential ripple effects on mental health.

In the context of measuring stress, the Perceived Stress Scale and the Chronic Stress Screening Scale demonstrated their mettle. Renowned for their efficacy, they meticulously gauged stress levels, shedding light on students' perceived stressors and their overall stress landscape, thereby painting a more complete picture.

Turning our attention to the profound contours of depression, our arsenal included the Self-Rating Depression Scale, Beck Depression Inventory, Depression, Anxiety, and Stress Scale-21, ACS-Depression (Affective Control Scale), and the Patient Health Questionnaire-9. These instruments formed a tapestry of assessment tools, each weaving a unique facet of the complex labyrinth of depression. Collectively, they facilitated a comprehensive evaluation of depressive symptoms, their severity, and the nuanced shifts over time.

In the realm of anxiety, the ACS-Anxiety, Self-Rating Anxiety Scale (SAS), Beck Anxiety Inventory, and Trait Anxiety Inventory became our compasses. Like seasoned explorers, they ventured into different corners of the anxiety landscape, unearthing the multifarious manifestations of this intricate emotion. The use of multiple measures allowed us to construct a holistic understanding of anxiety, an imperative in gauging the efficacy of interventions tailored to combat it.

Amidst these robust tools lie certain limitations that warrant attention. First and foremost is the specter of tool-specific bias. Each tool, with its distinctive lens, may inadvertently omit certain dimensions of a student's mental health. It's akin to examining a multifaceted gem with a singular facet, potentially overlooking important facets.

A second limitation emanates from the subjective nature of these assessments. The heavy reliance on self-reporting can introduce a subtle veil of bias. Students, influenced by societal expectations or their individual interpretations, may respond with subjectivity, thus tempering the precision of results.

Moreover, there exists variability in the sensitivity of these instruments. Although they are generally adept at detecting changes in mental health, the degree of sensitivity may waver based on individual disparities, cultural nuances, or other contextual factors.

Lastly, the timing and frequency of measurements in the constituent studies may not perfectly align with the interventions' duration and intensity. This temporal misalignment poses a potential challenge in accurately pinpointing the true effects of the interventions on the mental health landscape. These strengths and limitations of our chosen instruments collectively shaped the depth and scope of our research, providing a nuanced perspective on the mental wellbeing of university students.

### 2.8 Analytical decisions for meta-analysis

The results are presented in a forest diagram, showing the primary author, date of publication, sample size, individual effects using the Hedges index (g), and the overall effect with a 95% confidence interval, as well as the *p*-value associated with the statistic. A sensitivity analysis was performed, excluding studies containing individuals, duplicate data and atypical values. These results were then compared with the results obtained from the full meta-analysis.

For subgroup analysis or stratified analysis, we grouped studies according to the type of scale used for each mental health variable and performed separate meta-analyses within each group. This makes it possible to determine the effect size and variability within each subgroup, providing a more detailed understanding of the results.

In the sensitivity analysis process, certain studies were excluded to assess the robustness of our results. Exclusion criteria were based on study quality, data suitability, and the presence of duplicates. Studies that did not meet our inclusion criteria, such as those with deficient methodologies, insufficient data, or duplicates, were excluded. This exclusion was carried out to ensure that our analysis was based on high-quality studies and reliable data.

Definition of outliers: To define outliers, we applied specific statistical methods. We used measures such as Cook's D values, which assess the influence of individual data points on our results. When a Cook's D value indicated that a data point had a significant impact, it was considered for exclusion, or additional analyses were conducted to evaluate its impact on the robustness of our findings. This was done to ensure that outliers did not bias our conclusions. A meta-regression was performed to assess the influence of moderating variables according to the type of intervention prescription (frequency, duration, and volume). Finally, we assessed the risk of publication bias through the funnel-plot.

## 3 Results

### 3.1 Selection of studies

Extensive exploration of various databases was carried out, leading to the discovery of an initial set of 321 articles.

**Figure 1:**
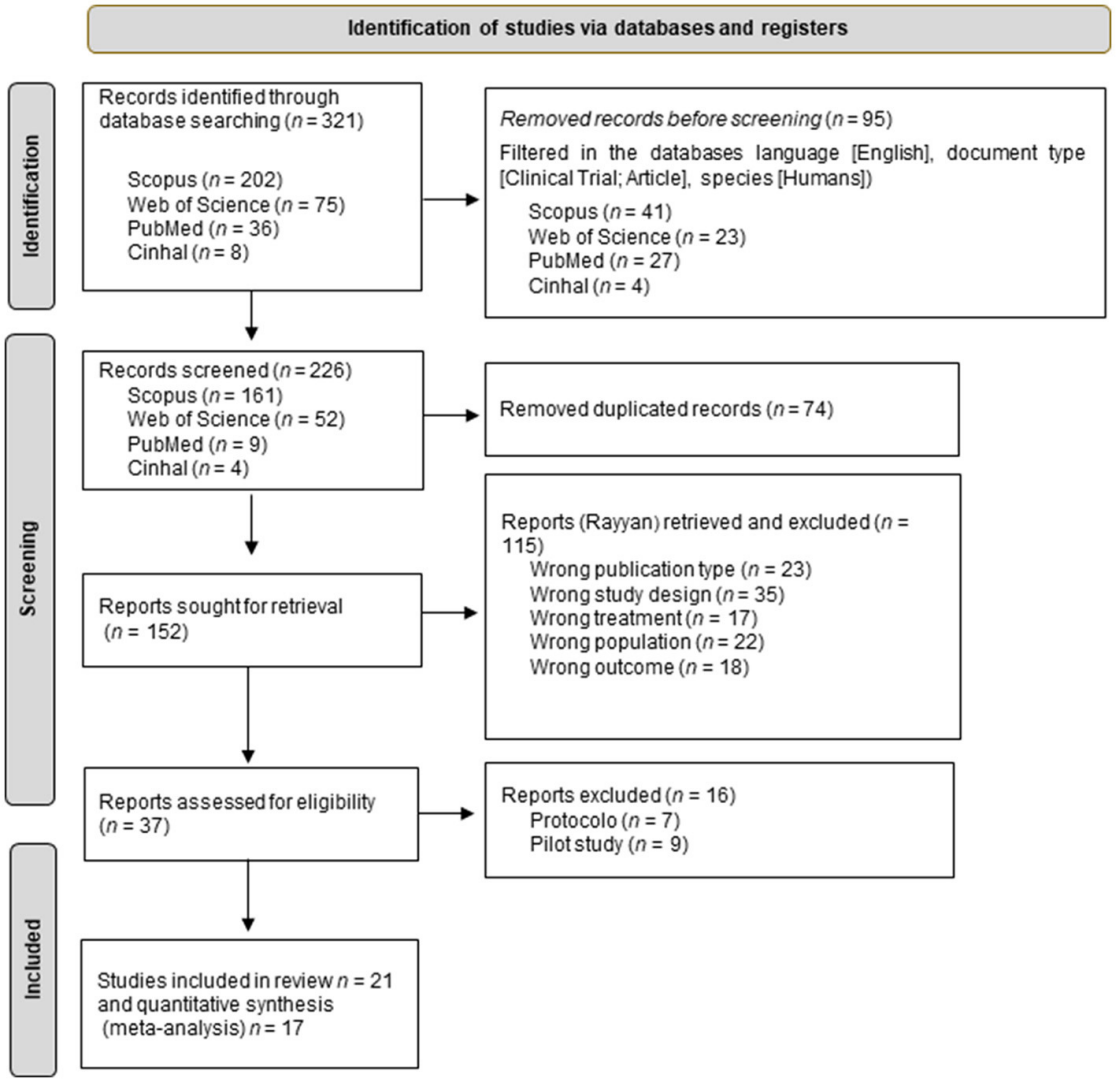
Flow diagram of the study selection process.

A total of 321 records were identified through database searching, with distribution across various databases: Scopus (*n* = 202), Web of Science (*n* = 75), PubMed (*n* = 36), and Cinhal (*n* = 8). Before the screening process, 95 records were removed, leaving 226 records to be screened. These 226 records underwent further filtering within the databases, narrowing them down based on criteria such as language [English], document type [Clinical Trial; Article], and species [Humans]. After this filtering process, 74 duplicate records were removed. Out of the remaining 152 reports sought for retrieval, 115 were retrieved but subsequently excluded due to reasons such as wrong publication type (*n* = 23), incorrect study design (*n* = 35), mismatched treatment (*n* = 17), inappropriate population (*n* = 22), and irrelevant outcome (*n* = 18). Finally, 37 reports were assessed for eligibility, out of which 16 were excluded, after completing this evaluation, the remaining articles were carefully analyzed to determine their eligibility. As a result, 21 articles were identified that met the established inclusion criteria (Figure 1) (24, 30–49), 17 of these studies underwent quantitative synthesis in the form of meta-analysis.

### 3.2 Methodological quality

A methodological quality assessment found that all the selected studies were identified as having a methodological quality considered “good” according to PEDro (Table 1).

**Table 1:** Methodological quality of the articles included.

**References**	**1**	**2**	**3**	**4**	**5**	**6**	**7**	**8**	**9**	**10**	**11**	**Total**
Lin and Mai (30)	Y	Y	N	Y	N	N	Y	N	Y	Y	Y	6
Galante et al. (31)	Y	Y	Y	Y	N	N	Y	Y	N	Y	Y	7
Flagobi et al. (24)	Y	Y	N	Y	N	N	Y	N	Y	Y	Y	6
Soriano Ayala et al. (32)	Y	Y	Y	Y	N	N	Y	Y	Y	Y	Y	8
Vorontsova-Wenger et al. (33)	Y	Y	Y	Y	N	N	Y	Y	Y	Y	Y	8
Moreno-Gómez et al. (34)	Y	Y	Y	Y	N	N	Y	Y	Y	Y	Y	8
De Vibe et al. (35)	Y	Y	Y	Y	N	N	Y	Y	Y	Y	Y	8
De Sousa et al. (36)	Y	Y	Y	Y	N	N	Y	N	Y	Y	Y	7
Erogul et al. (37)	Y	Y	Y	Y	N	N	Y	Y	N	Y	Y	7
Keng et al. (38)	Y	Y	N	Y	N	N	Y	Y	Y	Y	Y	7
Kuhlmann et al. (39)	Y	Y	N	Y	N	N	Y	Y	N	Y	Y	6
Van Dijk et al. (40)	Y	Y	N	Y	N	N	Y	Y	N	Y	Y	6
Song and Lindquist (41)	Y	Y	Y	Y	N	N	Y	N	Y	Y	Y	7
Dark-Freudeman et al. (42)	Y	Y	Y	Y	N	N	Y	Y	N	Y	Y	7
Deshpande et al. (43)	Y	Y	Y	Y	N	N	Y	N	Y	Y	Y	7
Li and Qin (44)	Y	Y	Y	Y	N	N	Y	Y	N	Y	Y	7
Morr et al. (45)	Y	Y	Y	Y	N	N	Y	N	Y	Y	Y	7
Xinyun et al. (46)	Y	Y	Y	Y	N	N	Y	Y	N	Y	Y	7
Gallego et al. (47)	Y	Y	Y	Y	N	N	Y	N	Y	Y	Y	7
Ritvo et al. (48)	Y	Y	Y	Y	N	N	Y	Y	y	Y	Y	8
Modrego et al. (49)	Y	Y	N	Y	N	N	Y	Y	Y	Y	Y	7

Items: 1, eligibility criteria; 2, random allocation; 3, concealed allocation; 4, baseline comparability; 5, blind subjects; 6, blind therapists; 7, blind assessors; 8, adequate follow-up; 9, intention-to-treat analysis; 10, between-group comparisons; 11, point estimates and variability; Y, Yes; N, No. The eligibility criteria item does not contribute to the total score.

### 3.3 Characteristics of the studies

In this systematic review, selected articles corresponding to randomized controlled clinical trials published in English in the last decade were included, with the following distribution of years: 2013 (35), 2014 (37, 39, 47), 2016 (30, 39), 2017 (38, 40), 2018 (31), 2020 (32, 43, 45), 2021 (36, 44, 46, 48, 49), 2022 (33, 42), and 2023 (24, 34). The countries of origin of the articles include Spain with four articles (32, 34, 47, 49), three articles from the USA (37, 42, 43), two from China (44, 46), two from Canada (45, 48), and one from each of the following countries: Taiwan (30), Australia (31), the United Kingdom (24), Switzerland (33), Norway (35), Brazil (36), Singapore (38), Germany (39), Netherlands (40), and South Korea (41).

In total, 4,463 individuals participated in the selected studies, of which, 1,741 were assigned to the experimental groups. The sample size in the 21 articles included in this systematic review ranged from 40 (36) to a maximum of 1,203 individuals (21). Sixteen articles selected in this systematic review primarily focused on depression, anxiety, stress, psychological distress and the regulation of emotions (33–37, 39–49), with stress being the most evaluated variable.

Mindfulness interventions mainly took place over 8 weeks. This was the case in 11 of the included articles that recorded this data (31, 37, 40–48). The longest intervention in the selected articles was 3 months (30), while the longest fol-low-up was carried out over 20 months (40). Full details of the articles selected in this review can be found in Table 2.

**Table 2:** Characteristics of the included studies.

**References**	**Country**	**Total sample size**	**Age mean**	**Control group**		**Experimental group**			**Variables**			**Results**
				**N**	**Intervention**	**N**	**Intervention**	**Time**	**Outcome**	**Measuring instrument**	**Assessments**	
Lin et al. (30)	Taiwan	55	–	27	MM: (1) students reviewed the chapter; (2) the teacher taught a chapter; (3) the teacher reviewed the chapter with the students for 5 min; (4) students then took an online formative assessment on the chapter; (5) students reviewed the results.	28	MM: (1) students practiced MM (10–20 min); (2) the teacher taught a chapter; (3) the teacher reviewed the chapter with the students (5 min); (4) students took an online FA about the chapter; (5) students reviewed the results.	3 months, 2 h a week	Formative assessment	8 chapters had 8 corresponding quizzes/ CAMS-R	At baseline (T0) and 12 weeks (T12)	IG T0 = 51.61 (24.64); T12 = 68.23 (24.92) CG T0 = 64.21 (19.52); T12 = 54.19 (29.08)
Galante et al. (31)	Australia	616	24	307	Regular support: access to comprehensive centralized support at the University of Cambridge Counseling Service, in addition to available support from the university and health services including the NHS.	309	Group and face-to-face skills training program based on the course book “Mindfulness: A practical guide to finding peace in a frenetic world”	8 weeks duration: 75 and 90 min	Psychological distress	CORE–OM	T0 = Baseline T1 = 8 weeks	IG T0 = 0.97 (0.51) T1 = 1.04 (0.54) EG T0 = 1.01 (0.54) T1 = 0.88 (0.53)
										WEMWBS		CG T0 = 48.61 (8.50) T1 = 46.87 IG T0 = 48.01 (8.58) (9.01) T1 = 49.61 (8.88)
Flagobi et al. (24)	United Kingdom	486	22.88	281	486 participants (Mage) completed a battery of measures at pre- and post-treatment. One class participated in the intervention (42%), whereas the other one did not (58%).	205	Brief online mindfulness-based intervention: The intervention included breathing meditation at the beginning of class, sharing of experiences, mini-lectures on mindfulness and daily practice.	28 consecutive days	Attention resources, developing a stronger sense of academic self-efficacy and improving the sense of belonging to a community	APSS	T0 = baseline T1 = 4 weeks (28 days)	Increased self-efficacy in learning regulation: IG (T0) = 2.99 (24.64) (T1) = 3.12 (24.92) *p <* 0.001 CG: T0 = 3.19 (19.52) T1 = 3.16 (29.08) Academic self-efficacy: IG: T0 = 2.99 (24.64) T1 = 3.07 (24.92) *p <* 0.01 CG T0 = 3.18 (19.52) T1 = 3.14 (29.08) *p =* 0.13
										MAIA		IG attention to bodily sensations: T0 = 2.76 T1= 2.98 *p <* 0.001
Soriano et al. (32)	Spain	51	20.94	25	Waiting list control group	26	Mindfulness training program called flow meditation: (i) mindfulness exercises from Kabat-Zinn; (ii) mindfulness techniques used in acceptance and commitment therapy; and (iii) exposure to and debate on metaphors and exercises used in Zen and Vipassana meditation	2 weekly hours for 7 weeks	Wellbeing	Healthy Lifestyle Questionnaire	T0 = baseline T1 = 7 weeks	IG: Tobacco T0 = 6.50 (2.98) T1 = 5.54 (2.26). Cannabis T0 = 5.54 (2.73) T1 = 4.92 (2.07). Alcohol T0 = 7.38 (3.03) T1 = 6.19 (2.11). Balanced diet T0 = 6.96 (1.48) T1 = 8.15 (1.40) Rest habits T0 = 6.92 (1.62) T1 = 8.08 (1.32). Externality T0 = 31.73 (7.15) T1 = 27.92 (5.78). Food consumption amount T0 = 16.27 (4.30) T1 = 12.81 (2.51). Snacking between meals T0 = 3.31 (1.12) T1 = 2.46 (0.51) Intake rate T0 = 7.08 (1.89) T1 = 6.08 (1.06)
										Lifestyle Questionnaire		
Vorontsova-Wenger et al. (33)	Switzerland	50	23.8	25	They received instructions on how to listen to the same neutral story every night. They were advised to do it lying down in a relaxed position.	25	Mindfulnes brief intervention-based stress reduction: 1 maintenance training session with the instructor, followed by daily individual practice of the BSM exercise for 30 min, for 8 weeks without any additional interaction with the instructor	8 weeks	Depression	BDI-2	T0 = baseline T1 = 8weeks	IG T0 = 14.16 (12.336) T1 = 6.00 (12.336) CG T0 = 0.84 (12.336) T1 = 10.60 (12.336) *p <* 0.001
									Anxiety	STAI-Y		IG T0 = 31.40 (12.336) T1 = 27.10 (14.952) CG T0 = 38.72 (12.374) T1 = 39.36 (11.909) *p <* 0.001
									Stress	PSS		IG T0 = 33.96 (3.195) T1 = 18.12 (6.064) *p <* 0.001
									Academic performance	Mean grade		EG T0 = 4.74 (0.396) T1 = 5.28 (0.491) CG T0 = 4.71 (0.387) T1 = 4.76 (0.372)
Moreno et al. (34)	Spain	137	19.94	68	–	69	Program's implementation (MK-A): content (1) respiration, (2) my body and me, (3) my thought, (4) how I feel, and (5) contemplation	2 information sessions (1 h per session)	Dispositional mindfulness	FFMQ	T0 = baseline T1 = 2 weeks	T0 IG = 118.90 (14.84) CG = 122.35 (14.18) T1 IG = 124.26 (16.77) CG = 119.00 (13.77)
									Mental health	MH-5		T0 IG = 16.50 (3.97) CG = 14.00 (4.24)
									Regulation of emotions	SVDERS		T0 IG 145.81 (18.62) CG = 145.83 (19.50) T1 IG = 53.76 (18.57) CG = 56.25 (16.43)
De Vibe et al. (35)	Norway	288	23	144	–	144	MBRS programme: (1) physical and mental exercises to increase participant mindfulness of experiences in the present moment, (2) didactic teaching on mindfulness using a course manual and CDs for home practice, and (3) a group process to facilitate reflections on practicing mindfulness at home and during classes	7 weeks: 6 weekly sessions, 1.5 h each, one 6-h session in week 7, and 30 min of daily mindfulness practice at home	Psychological distress	GHQ1	T0 = baseline T1 = 7 weeks	IG T0 = 12.4 (6.0) T1 = 9.2 (4.0) CG T0 = 13.0 (6.2) T1 = 13.2 (6.1)
									Stress	PMSS		IG T0 = 18.9 (6.9) T1 = 18.4 (6.8) CG T0 = 19.5 (7.0) T1 = 20.3 (7.4)
									Subjective wellbeing	SWB		IG T0 = 6.3 (1.8) T1 = 6.8 (1.4) CG T0 = 6.4 (1.8) T1 = 6.1 (1.8)
									Mindfulness	FFMQ		IG T0 = 20.5 (3.8) T1 = 21.9 (3.6) CG T0 = 20.4 (3.9) T1 = 20.7 (4.0)
De Sousa et al. (36)	Brazil	40	24,15	20	AC groups listened to an audio that contains educational health information for about 15 min and coloring 30 min a day.	20	MT: During the 3 days of intervention, the same audio of 30 min for meditation practice, a standard mindfulness sitting meditation practice	Short mindfulness training: 3 sessions of 30 min (90 min total)	Perceived stress	PSS	T0 = baseline T1 = 7 weeks	T0 Baseline CG = 30.50 (7.39) IG = 30.60 (6.85) T1 CG = 28.35 (6.65) IG = 24.25 (8.18)
									Anxiety	STAI		T0 Baseline CG = 38.65 (9.43) IG = 39.70 (7.03) T1 CG = 38.65 (9.82) IG = 35.20 (7.28)
									State mindfulness	FFMQ		T0 Baseline CG = 74.50 (13.02) IG = 71.50 (9.38) T1 CG = 69.95 (17.54) IG = 76.70 (12.09)
Erogul et al. (37)	USA	58	23.5	30	Control group did not receive any intervention during the 8-week study period	28	Standard MBSR program. Homework included individual sessions of daily meditation for 20 min	8 weeks and weekly class duration 75 min	Stress	PMSS	T0 = baseline T1 = 8 weeks t2 = 6 moths after intervention	PSS achieved significant reduction (3.63, *P =* 0.03), 95% CI (0.37, 6.89), but not at 6 months post-study (2.91, *P =* 0.08), 95% CI (−0.37, 6.19).
									Resilience	RS		The study did not show a difference in SR after the intervention.
Keng et al. (38)	Singapur	171	20.19	114	Reappraisal or suppression: Audio instruction of the assigned strategy for dealing with negative emotions (10 min.) Reevaluation group *=* trained to reformulate the meaning of an emotional event to reduce emotional impact and engage in an exercise. Suppression group *=* suppress both the experience and expression of emotions.	57	Mindfulness: Recording thoughts and emotions as they are without judging them; included an experiential mindfulness exercise.	1.5-h experimental session	Regulation of emotions	State sadness	T0 = baseline T1 = post Intervention	Suppression vs. mindfulness 2.3 (1.07) *p =* 0.03. Marginally significant trend (*p =* 0.052, d = −0.21)
Kuhlmann et al. (39)	Germany	80	23.39	17	No treatment	63	MediMind: Booklet attached to participants containing the content of each training session and instructions for practice assignments. Two other physical education coaches conducted Autogenic Training).	5 weeks for 90 min	Psychological distress	BSI	T0 = baseline T1 = 3 weeks T2 = follow 1 year	In the BSI, a significant interaction effect was evident (*p =* 0.002, partial η^2^ = 0.382).
									Stress	GSI		T0 IG = 55 ± 0.44 AT = 0.57 ± 0.37 CG = 0.56 ± 0.39 T1 IG = 0.54 ± 0.52 AT = 0.58 ± 0.44 CG = 0.49 ± 0.40 T2 IG = 43 ± 0.34 AT = 0.66 ± 0.54 CG = 34.55 ± 0.41
										SSCS		T0 IG = 21.29 ± 8.86 AT = 19.44 ± 7.93 CG = 22.18 ± 7.49 T1 IG = 18.58 ± 8.09 AT = 18.81 ± 8.15 CG = 20.35 ± 8.94 T2 IG = 20.42 ± 7.81 AT = 22.72 ± 8.79 CG = 19.47 ± 7.16
Van Dijk et al. (40)	Netherlands	167	23,3	84	CAU	83	MBRS: 2-h sessions were held in different academic periods	8 sessions 2 h and 8 weeks home practice	Psychological distress	BSI	T0 = baseline T1 = 3 month T2 = 7, T3 = 12, T4 =15, T5 = 20 months	IG T0 = 0.38 (0.26); T1 = 0.31 (0.26); T2 = 0.30 (0.23); T3 = 0.32 (0.25), T4 = 0.31 (0.26); T5 = 0.30 (0.23); CG T0 = 0.42 (0.29); T1 = 0.36 (0.28); T2 = 0.45 (0.38); T3 = 0.40 (0.36); T4 = 0.28 (0.21); T5 = 0.37 (0.32)
									Mental health	MHC-SF		IGT0 = 44.9 (10.6); T1 = 46.7 (10.0); T2 = 49.5 (8.9); T3 = 48.4 (10.6); T4 = 49.4 (11.5); T5 = 51.4 (11.6); CG T0 = 45.2 (8.9); T1 = 46.1 (9.1); T2 = 44.4 (10.4); T3 = 42.3 (11.7); T4 = 46.7 (9.6); T5 = 46.2 (10.2)
									State mindfulness	FFMQ		MBSR T0 = 131.3 (14.7); T1 = 134.0 (13.7); T2 = 135.0 (14.1); T3 = 135.2 (14.7); T4 = 134.0 (18.4); T5 = 135.6 (17.9); CG T0 = 128.5 (14.0); T1 = 127.7 (13.9); T2 = 125.9 (14.9); T3 = 127.8 (15.8); T4 = 131.6 (15.0); T5 = 127.7 (16.6)
Song and Lindquist (41)	Korea	44	19.5	21	No treatment	23	MBSR group practiced mindfulness meditation: elements of yoga, sitting, walking, breath-work, body scan, and eating meditation.	8 days and each session was 2 h per week for 8 weeks	Depression	DASS-D	T0 = baseline T1 = 8 weeks	IGT0 IG = 8.3 (5.1) CG 8.6 (8.9) T1 IG = 4.1 (4.0) CG = 8.5 (7.6) F = 10.99 *p =* 0.002
									Anxiety	DASS-A		IGT0 IG = 6.7 (12.6) CG 5.9 (6.3) T1 IG = 2.8 (4.1) CG 5.9 (7.4) F = 5.61 *p =* 0.023
									Stress	DASS-S		T0 IG = 34.5 (12.5) CG 30.0 (12.2) T1 IG = 7.4 (4.9) CG = 13.7 (8.9) F = 15.31 *p =* 0.001
									The mindfulness attention	MAAS		T0 IG = 69.8 (10.6) CG = 77.7 (16.3) T1 IG = 80.6 (11.3) CG = 79.0 (12.6) F = 5.03 *p =* 0.010
Dark-Freudeman et al. (42)	USA	77	20.92	26	No treatment; coloring	23	MBI: Instructed to listen to the traditional technique for 15 min each day during the week	4 weekly intervention sessions of 30 min	Anxiety	STAI	T0 = baseline T1= 4 weeks	IG T0 = 41.70 (10.90) T1 = 38.00 (10.41) CG T0 = 45.82 (10.88) T1 = 46.39 (12.00)
									Stress	PSS		IG T0 = 21.35 (5.61) T1 = 14.70 (4.39) CG T0 = 20.86 (6.69) T1 = 20.32 (5.75)
									State mindfulness	FFMQ		IG T0 = 125.87 (13.84) T1 = 138.43 (12.57) CG T0 = 119.54 (14.49) T1 = 117.00 (13.95)
Deshpande et al. (43)	USA	115	-	-	-	90	MBRS 2.5 h for 8 weeks, with a 6-h mindfulness day retreat held after the sixth session.	8 weeks	Stress	PSS	T0 = baseline T1 = 8 weeks	IG T0 = 2.84 (0.69) T1 = 2.41 (0.68)
									State mindfulness	FFMQ		IG T0 = 2.87 (0.42) T1 = 3.41 (0.50)
									Psychological distress	CCAPS-34		IG T0 = 1.29 (0.55) T1 = 0.97 (0.50)
Li et al. (44)	China	106	21	66	No treatment	62	MBSR	8 weekly, 2.5 h group classes	Regulation of emotions	ACS	T0 = baseline T1 = 8 weeks	T0 IG = 166.50 (25.07) CG = 155.86 (14.20) T1 IG = 134.95 (29.07) CG = 149.68 (26.38)
									Depression	ACS-depression		T0 IG = 34.05 (8.54) CG = 30.5 (5.71) T1 IG = 26.8 (7.64) CG = 28.64 (8.94)
									Anxiety	ACS-anxiety		T0 IG = 51.9 (11.19) CG = 46.93 (5.58) T1 IG = 39.6 (9.87) CG = 44.82 (9.56)
									State mindfulness	MAAS		T0 IG= 56.15 (6.74) CG = 56.93 (7.41) T1 IG = 68.3 (5.93) CG = 57.89 (7.87)
El Morr et al. (45)	Canada	160	22,55	80	No treatment	80	TCC-mindfulness consisted of a web-based platform comprising (1) 12 psychoeducation-based modules; (2) anonymous peer-to-peer discussion forums; and (3) 20-min professional-guided video conferencing	8 weeks	State mindfulness	FFMQ	T0 = baseline T1 = 8 weeks	Significant increase in mindfulness score (β = 4.84, *p =* 0.02)
									Stress	PSS		There was no statistically significant difference in perceived stress for CVS (β = 0.64, *p =* 0.48)
									Anxiety	BAI		Decreased anxiety score (β = −4.82, *p =* 0.006)
									Depression	PHQ9		Statistically significant reduction among depressed groups (β = −2.21, *p =* 0.01).
Yuan et al. (46)	China	1,203	–	1,100	No treatment	103	Mindfulness: 64 online groups and 39 offline groups.	8 weeks face-to-face and online mindfulness	Anxiety	SAS	T0 = baseline T1 = 8 weeks	SAS, SDS scores (5.57, 5.31, 3.99; 4.88, *p <* 0.01)
									Depression	SDS	
Gallego et al. (47)	Spain	125	20,07	84	Education intervention group (42 students) or the control group (42 students).	41	MBCT: Sessions were adapted to a weekly 1-h session, maintaining the main structure	8 weeks, weekly 1-h session	Depression	DASS-D	T0 = baseline T1 = 8 weeks	IG T0 = 4.75 (3.55) T1 = 2.90 (2.50) *p =* 0.053
									Anxiety	DASS-A		IG T0 = 4.47 (3.78) T1 = 3.46 (2.41) *p =* 0.480
									Stress	DASS-S		IG T0 = 7.95 (3.89) T1 = 5.70 (2.75) *p =* 0.006
Ritvo et al. (48)	Canada	154	23.1	78	No treatment	76	Mindfulness (1) educational and mindfulness video modules, (2) anonymous peer-to-peer discussions, and (3) anonymous, group-based, professionally guided, 20 min. videoconferences	8 weeks	Anxiety	BAI	T0 = baseline T1 = 8 weeks	IG β = −2.06, *p =* 0.31 CG β = −2.32, *p =* 0.27) no differences
									Stress	PSS		There was a significant difference for the PSS: β = −2.31, *p =* 0.03;)
									State mindfulness	FFMQ-SF		IG β = 1.33, *p =* 0.43;.
Modrego et al. (49)	Spain	280	21.95	94	Relaxation	93 MBP y 93 MBP+VR	MBP that proved efficacy for reducing generalized anxiety disorder symptomatology offered as an extra-curricular activity/MBP + VR = reduced to 75 min	6 weeks 90-min group sessions with 15 or 16 participants per subgroup in MBPP + VR = 75 min	Stress	PSS	T0 = baseline T1 = 6 weeks	T0 IG = 19.73 (4.02) IG+VR = 19.81 (4.41) CG = 19.35 (3.63) T1 IG = 15.33 (4.50) IG+VR = 15.75 (4.51) CG = 17.73 (4.52)
									Anxiety	STAI		T0 IG = 26.27 (4.73) IG+VR = 26.15 (5.23) CG = 25.16 (4.30); T1 IG = 20.29 (5.41) IG+VR = 21.37 (5.41) CG = 23.54 (5.42)
									State mindfulness	FFMQ-SF		T0 IG = 120.14 (9.48) IG+VR = 120.05 (10.73) CG = 121.00 (8.37) T1 IG = 135.48 (10.90) IG+VR = 133.39 (10.92) CG = 127.17 (10.98)

T, time; CG, control group; IG, intervention group; MM, mindfulness meditation; CAMS-R, Cognitive and Affective Mindfulness Scale-Revised; NHS, National Health Service; CORE–OM, Clinical Outcomes in Routine Evaluation Outcome Measure; WEMWBS, Warwick-Edinburgh Mental Wellbeing Scale; APSS, Academic Perceived Self-Efficacy Scale; PT, total score; MAIA, The Multidimensional Assessment of Interoceptive Awareness; SSC-CUO, Scale of Sense of Community in University Online Courses; BDI-2, French version of the Beck Depression Inventory; STAI-Y, French “Form Y” version of the State-Trait Anxiety Inventory; PSS, Perceived Stress Scale; BSM, body scan meditation; MK-A, MindKinder adult version program; FFMQ, Five Facets Mindfulness Questionnaire; MH-5, Mental Health-5; SVDERS, Spanish Version of the Difficulties in Emotion Regulation Scale; GHQ12, General Health Questionnaire; PMSS, perceived medical school stress; SWB, subjective wellbeing; AC, active control; MT, mindfulness training; STAI, Trait Anxiety Inventory; RS, Resilience Scale; BSI, Brief Symptoms Inventory; GSI, Global Severity Index; SSCS, Chronic Stress Screening Scale; CAU, clerkships as usual; MHC-SF, Mental Health Continuum-Short Form; DASS-D, Depression, Anxiety and Stress Scale-21; MAAS, The Mindfulness Attention Awareness Scale; CCAPS-34, Counseling Center Assessment of Psychological Services; MBSR, Mindfulness-Based Stress Reduction; ACS, Affective Control Scale; BAI, Beck Anxiety Inventory; PHQ9, Patient Health Questionnaire-9; SAS, Self-Rating Anxiety Scale; SDS, Self-Rating Depression Scale; MBCT, Mindfulness-Based Cognitive Therapy; MBP, mindfulness-based programme; MBP: mindfulness-based programme + virtual reality.

### 3.4 Study results

Of the 21 articles included in this review, 19 demonstrated that mindfulness interventions had a significant positive effect on students' mental health (24, 30–33, 37–49). Table 2 shows the details of these studies. In particular, significant results (*p* < 0.01) were observed on stress management, which was the most frequently evaluated variable. These results were mainly obtained using the Perceived Stress Scale (PSS) (33, 36, 37, 42, 43, 48, 49). Only one study using this measurement tool did not obtain significant results (F = 5.38, *p* = 0.021) (35). A significant impact of mindfulness intervention on stress was also found when measured with the DASS-S. This was true in Song and Lindquist (F = 10.99, *p* = 0.002) (41) and Gallego et al. (F = 5.91, *p* = 0.004) (47). Kuhlmann et al. did not find there to be a significant effect (*p* = 0.251) when using the Chronic Stress Screening Scale (SSCS) (39).

The second most studied variable was mindfulness. It was evaluated with the Mindful Attention Awareness Scale (MAAS) in two studies and found to be significant: Song and Lindquist (41) and Li et al. (44) found a significant positive effect with *p* = 0.01 and *p* = 0.001 respectively. The authors of nine articles investigated the effectiveness of improving mindfulness in university students, measuring it with the Five Facets Mindfulness Questionnaire (FFMQ) (34–36, 40, 42, 43, 45, 48, 49). Eight of these studies showed a significant positive increase in mindfulness despite the difference in duration of the interventions. Modrego et al. (49) found significant results for a six-week intervention (*p* < 0.001), while De Vibe et al. (35) and De Sousa et al. (36) found significant results with 7-week interventions (*p* < 0.001). Deshpande et al. (43) and Morr et al. (45) found a significant increase in FFMQ scores (*p* = 0.04 and *p* = 0.02 respectively) after 8 weeks of intervention. However, Ritvo et al. (48) found no significant changes in the same period (*p* = 0.41). Finally, Van Dijk et al. (40) highlighted that the intervention group (IG) showed a significant improvement compared to the care-as-usual group both after 3 months of intervention and 20 months of follow-up (*p* = 0.04).

All the investigations that included the depression variable in the analysis found that an eight-week intervention was successful. Yuan et al. (46) and Gallego et al. (47) reported significant findings (*p* > 0.01) using the Self-Rating Depression Scale (SDS) for both virtual (23) and face-to-face interventions (24). Vorontsova-Wenger et al. (33) used the French version of the Beck Depression Inventory (BDI-2), finding significant differences (*p* < 0.001) intra and intergroup with scores for IG in pre-intervention 14.16 (12.336) vs. post 6.00 (12.336) and for pre-intervention CG 0.84 (12.336) vs. post 10.60 (12.336). Using the Depression, Anxiety and Stress Scale-21 (DASS-D), Song and Lindquist (41) recorded GI differences with respect to the control group (F = 10.99 *p* = 0.002); Van Dijk et al. (40) found positive changes when using the ACS-depression Affective Control Scale (ACS) (F = 9.34 *p* = 0.004), as did Morr et al. (45) with the Patient Health Questionnaire-9 (PHQ9) (β = −2.21, *p* = 0.01).

Anxiety was the object of study of 10 articles, of which four were evaluated using the State-Trait Anxiety Inventory (STAI-Y) obtaining differences in favor of the GI. Vorontsova-Wenger et al. (33) reported statistical significance (*p* > 0.001) at 30 min of weekly intervention for 8 weeks, as did De Sousa et al. (36) with 30 min of weekly intervention for 3 weeks (*p* > 0.001), Dark-Freudeman et al. (42) with 30 min of weekly intervention for 4 weeks (*p* = 0.005) and Modrego et al. (49) with 90 min per week for 6 weeks (*p* = 0.007). With the Beck Anxiety Inventory (BAI), divergent results were found with interventions of the same duration: 20 min per week for 8 weeks. Morr et al. (45) reported a significance of *p* = 0.006 while Ritvo et al. (48) found no differences (*p* = 0.31).

Studies measuring anxiety with the Depression, Anxiety and Stress Scale-21 DASS (DASS-A) examined interventions of 8 weeks, but had contradictory results: Song and Lindquist (41) found significant changes (*p* = 0.023) at 120 min per week, while Gallego et al. (47) had significant findings with interventions shorter than 60 min per week (*p* = 0.480). During this same period, Li et al. (44) used the ACS-anxiety and had favorable results (*p* = 0.001) for 150 min per week. This was in opposition to Yuan et al. (46) who used the Self-rating Anxiety Scale SAS and had no significant results (*p* = 0.01) with shorter sessions (30 min). This indicates that longer sessions were more effective, regardless of how many weeks the intervention takes place over.

Another variable addressed was psychological distress. Different scales were used. The Brief Symptom Inventory (BSI) (39, 40) was used and both 90 min (39) of intervention for 5 weeks (*p* = 0.002) and 120 min (40) per week for 8 weeks (*p* = 0.03) were found to be statistically significant. Over the same period, Galante et al. found that 90-min sessions were effective using the Warwick-Edinburgh Mental Wellbeing Scale (*p* = 0.001) (31). Deshpande et al. (43) used the Psychological Distress Counseling Center Assessment of Psychological Services (CCAPS-34) and found that sessions of 150 min had a significant effect (*p* = 0.001), while De Vibe et al. (35) found a more statistically significant result (*p* = 0.021) for 7 weeks of management for 90 min using the General Health Questionnaire.

In addition, other related variables in the study, such as attention and cognitive awareness (CAMS-R), were analyzed but no significant differences were found between the groups. Although the regulation of emotions was evaluated as a key factor in the study, heterogeneity was observed in the conditions, measurements and instruments used, which excluded it from the meta-analysis. However, significant improvements in trait emotional intelligence were found in favor of the experimental group, with a small effect size (η^2^ = 0.045 *p* = 0.04) (34). Regarding the regulation of specific emotions, it was observed that the mindfulness group showed significantly greater participation (mean difference = 5.33) compared to the re-evaluation group (mean difference = 4.61, *p* < 0.01) and the suppression group (M = 3.86, *p* < 0.001) in the regulation of sadness. However, despite the positive effect of mindfulness on decreasing sadness, the suppression approach was found to be associated with significantly lower average sadness over the entire regulation period compared to mindfulness (*p* = 0.002) (38). These results indicate that the mindful-ness intervention had a positive impact on affective control in the experimental group. While no significant differences were found in the regulation of emotions in general, improvements in trait emotional intelligence were observed in favor of the experimental group. In addition, regarding regulating specific emotions, mindfulness was found to be more effective in regulating sadness compared to reappraisal and emotional suppression. However, more research is needed to fully understand the effects of the intervention on emotional regulation.

The scientific literature has investigated the impact of mental wellbeing on academic performance, highlighting its relationship with a healthy lifestyle and the reduction of behaviors associated with negative habits (32). In addition, the results indicate that experimental group participants, compared to the control group, experienced significant improvements in various aspects of their academic performance. These included a greater sense of influence over course activities (F = 9.628, *p* < 0.005), better self-regulation of attention (F = 19.133, *p* < 0.001), greater academic self-efficacy (F = 9.220; *p* < 0.005) and, in particular, greater self-efficacy in learning regulation (F = 12.942; *p* < 0.001) (24). In addition, a positive relationship between mental wellbeing and specific academic performance was observed (33).

The review of 21 articles consistently revealed positive outcomes of mindfulness interventions in university students on mental health. Overall, a significant improvement was observed in various aspects, including stress management, mindfulness, reduction of depression and anxiety, and decreased psychological distress. Additionally, improvements in academic performance and a sense of wellbeing were reported.

Despite these positive results, there was some heterogeneity among the studies, indicating that effects were not uniform across all cases. Variations in intervention duration and measurement scales used contributed to this heterogeneity. However, the majority of studies demonstrated consistent mental health benefits, regardless of the intervention duration.

These findings suggest that mindfulness interventions have a significant and positive impact on the mental health of university students. The intervention consistently contributed to stress reduction, increased mindfulness, and overall improved mental wellbeing, making them valuable for enhancing both mental health and academic performance among students.

The selected studies have several limitations that should be considered when evaluating the robustness of the findings. Some of the common limitations include:

Heterogeneity in Intervention Duration: The studies varied in the duration of mindfulness interventions, which could influence the magnitude of observed effects. Some studies had short-duration interventions, while others extended over several weeks. This variability in duration may complicate result comparisons and generalization of findings.

Diversity in Measurement Scales: Studies used different scales and measures to assess the same constructs such as stress, anxiety, and mindfulness. This could introduce some variability in results and make direct comparisons between studies challenging.

Varied Sample Sizes: Some studies had small sample sizes, which can limit the generalizability of the results to larger populations. Small samples can also increase the risk of bias and limit the robustness of findings.

Selection Bias: In some studies, participants who chose to engage in mindfulness interventions might have had different personal characteristics compared to those who did not participate. This could introduce selection bias that influences the results.

Lack of Active Control Groups: Some studies may lack active control groups, making it challenging to determine whether the observed effects are specific to mindfulness interventions or simply a result of the additional attention participants receive.

Despite these limitations, the overall results point toward positive effects of mindfulness interventions on the mental health of university students. However, it is crucial to consider these limitations when interpreting the results and when planning future research in this field.

### 3.5 Meta-analysis

In the present meta-analysis, a total of 17 articles were included with the aim of synthesizing findings related to various mental health variables. Each mental health variable was analyzed and considered separately in the study.

#### 3.5.1 Stress

The first analysis was carried out with the aim of synthesizing existing studies on the effect of mindfulness strategies on stress in university students. For the evaluation of heterogeneity, the I-square statistic was used, which yielded an approximate value of 59%, indicating moderate to high heterogeneity. Likewise, the Q value of 26.595 with 11 degrees of freedom, and a value of *p* = 0.005 was estimated. Using an alpha significance level of 0.100, the null hypothesis that the true effect size is the same in all studies can be rejected. As for Tau-squared and Tau, the following values were obtained: Tau-squared, which represents the variance of the true effect sizes, was 0.024 in d units, while Tau, which is the standard deviation of the true effect sizes, was 0.155 in d units. For all the above, the random-effects model was used for the analysis.

The overall mean effect size was −0.342, with a 95% confidence interval ranging from −0.464 to −0.220. To test the null hypothesis (the mean effect size is zero), the Z-value was used. The result obtained was −5.498, with a *p* value of <0.001. Using an alpha significance level of 0.050, the null hypothesis can be rejected and we can conclude that in populations comparable to those of the analysis, the mean effect size is not equal to zero. Finally, the prediction interval was estimated to be between −0.713 and 0.029. This implies that the true effect size, in 95% of all comparable populations, lies within this range.

**Figure 2:**
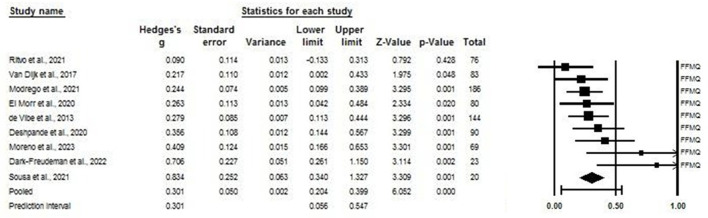
Forest plot: effect of mindfulness on the stress of university students according to the scale used. The black box represents the point estimate for the respective study, while the size of the box rep-resents the population size. The horizontal line is the 95% CI. The diamond-shaped figure represents the estimated point of the mean difference.

With the analysis of subgroups (Figure 2) for each of the stress measurement tools showed a reduction with a mean size (0.317) with a prediction interval between −0.425 and 0.029. Studies using the Depression, Anxiety, and Stress Scale-21 DASS-S (41, 47) showed an effect size of 0.56 (Hedges' g) with 95% CI between −0.862 and −0.260 *p* = 0.000, while those using the PSS (33, 36, 37, 42, 43, 48, 49). Were synthesized in an effect size of 0.325 (Hedges' g) with a 95% CI between −0.451 and −0.189 *p* = 0.000. The only article that examined stress using the Chronic Stress Screening Scale (SSCS) (39) had a small effect size (Hedges' g = 0.144 *p* = 0.249).

Depressive symptoms, as measured by the GDS, significantly decreased over time (*p* = 0.007, Cohen's d = 0.39). Scores for the intervention group (IG) were significantly lowered to 0.55 ± 0.2, compared with 0.8 ± 3.9 for the basic test group (*p* = 0.028) (43). Results in favor of the computer-based cognitive training (CBCT) group demonstrated changes in depression scores, with small effect sizes ranging from 0.21 to 0.36 (45).

A group treatment of cognitive rehabilitation and cognitive-behavioral treatment for early dementia (CORDIAL) is feasible in a clinical routine setting and demonstrated antidepressant effects in the CBT IG compared with regular care (40). A CBT program with musical and artistic stimuli achieved changes at the end of twenty-four sessions compared to initial values (*p* = 0.013) (36). Likewise, in 12 weeks, Keng et al. (38) used the Beck Depression Inventory to verify that a lower level of depression can be reached for the IG compared to the control group (CG) (*p* = 0.015). This research highlights that, in patients with AD, CBT can improve initiative and stabilize memory, while non-cognitive treatments can improve psychosocial aspects.

However, among the investigations that used the MADRS scale for the evaluation of depression, there were no significant differences in the group x time interaction. Among these is the Norwegian study (35), where the regression coefficient in the GI is −1.31 (0.83) and a non-significant interaction is reported (*p* = 0.34), and the French study (37) (*p* = 0.916) (Table 2).

This analysis supports the effectiveness of mindfulness strategies in reducing stress among university students. Furthermore, a significant decrease in depressive symptoms was observed, particularly in interventions that included computer-based cognitive training, cognitive rehabilitation, and cognitive-behavioral treatment, with effect sizes ranging from small to moderate. The effect size for stress reduction (−0.342) suggests a significant decrease in stress levels among university students. In real life, this means that mindfulness strategies can be effective in helping students better manage stress related to their studies and other concerns, which, in turn, can enhance their overall wellbeing and academic performance.

#### 3.5.2 Anxiety

The results of the meta-analysis from studies controlling anxiety in students using mindfulness revealed a mean effect size of −0.309, with a 95% confidence interval training from −0.417 to −0.201. When performing the Q test, a value of 14.542 was obtained with 9 degrees of freedom and a value of p = 0.104. The I-square statistic showed moderate heterogeneity in the included studies, with a value of 38%. In addition, the Tau-squared, which represents the variance of true effect sizes, was calculated at 0.011. The prediction interval is between −0.579 and −0.039. These analyses were carried out using the random-effects model. With this, it was estimated that the average effect of the intervention on reducing anxiety in students decreased by −0.309. However, it should be noted that there is some uncertainty in this estimate and the actual effect size could be within the range of −0.417 to −0.201.

**Figure 3:**
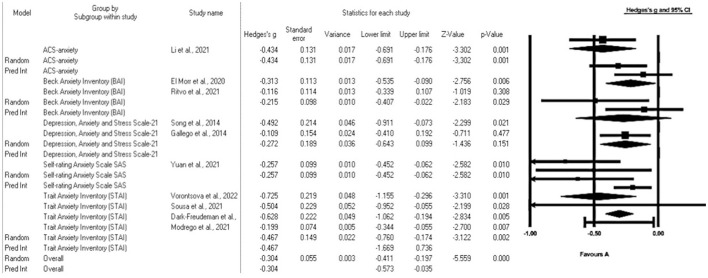
Forest plot: subgroup analysis of the effect of mindfulness on the anxiety of university students according to the scale used. The black box represents the point estimate for the respective study, while the size of the box represents the population size. The horizontal line is the 95% CI. The diamond-shaped figure represents the estimated point of the mean difference.

The analysis of subgroups according to the measurement scales found a median effect size for study with ACS anxiety Hedges' g = 0.434 p = 0.001 (44) and Trait Anxiety Inventory (STAI) Hedges' g = 0.467 p = 0.002 (30, 32, 38, 45); and a small effect size for those who measured with the BAI Hedges' g = 0.215 p = 0.029 (41, 44); Depression, Anxiety, and Stress Scale-21 DASS-A Hedge's g = 0.272 p = 0.151 (41, 47) and Self-Rating Anxiety Scale SAS Hedges' g = 0.257 p = 0.01 (46) (Figure 3).

The subgroup analysis, irrespective of the anxiety measurement scale employed, consistently showed the effectiveness of mindfulness strategies in reducing anxiety. This is underlined by a spectrum of effect sizes, ranging from moderate to small. In this context, the “effect size” serves as a measure of the magnitude of the difference in anxiety levels between the group that received mindfulness strategies and the control group, offering a clear indicator of the significance and clinical relevance of these anxiety reduction strategies. While moderate effect sizes signify a substantial impact, small effect sizes indicate a more modest yet still beneficial effect on anxiety reduction. Consequently, mindfulness strategies prove to be advantageous in diminishing anxiety among university students, enhancing their capacity to tackle both academic and emotional challenges.

#### 3.5.3 Depression

In this analysis, the random-effects model was used to examine changes in depression in undergraduate students. Since the heterogeneity Q test, which evaluates the null hypothesis that all studies share a common effect size, yielded a value of 6.977 with 5 degrees of freedom and p = 0.222. The I-squared statistic, which indicates the proportion of variability in observed effects reflecting variability in true effects rather than sampling error, was 28%. Tau-square and Tau were calculated to assess the variability of true effect sizes. The Tau-square, which represents the variability between true effects, was 0.008 in g units, while the Tau, which is the standard deviation of the true effect sizes, was 0.089 in g units.

We selected six studies and assumed that these studies represent a random sample of a wider universe of depression research. The mean size of the effect obtained was −0.382, indicating a significant decrease in symptoms of depression with a 95% CI ranging from −0.517 to −0.246. The Z-value, which evaluates the null hypothesis that the mean effect size is zero, was −5.523 with p < 0.001. Using an alpha significance level of 0.050, the null hypothesis can be rejected and a conclusion can be drawn that, in the universe of populations comparable to the analysis, the mean effect size is not zero, indicating that there is a significant impact on the reduction of depression symptoms.

**Figure 4:**
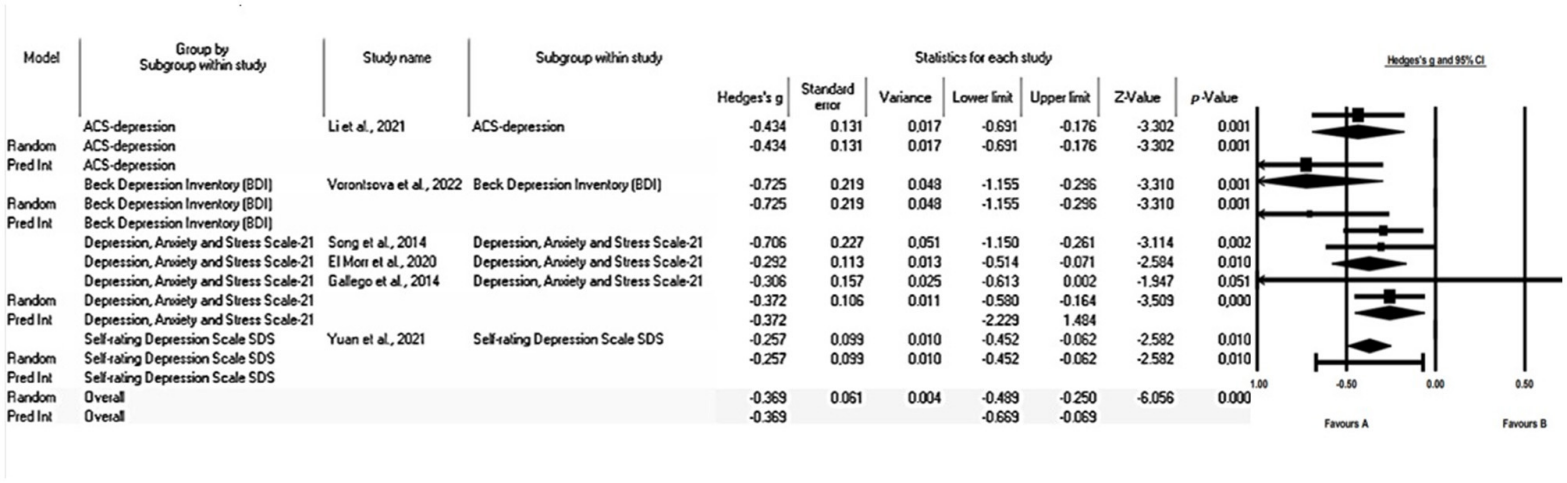
Forest plot: subgroup analysis of the effect of mindfulness on depression in college students according to the scale used. The black box represents the point estimate for the respective study, while the size of the box represents the population size. The horizontal line is the 95% CI. The diamond-shaped figure represents the estimated point of the mean difference.

A subgroup analysis based on the different measurement scales revealed effect sizes of different magnitudes (Figure 4). In particular, we found a median effect size (Hedges' g = 0.725 p = 0.001). For the study that used the BDI scale (33). On the other hand, a small effect size was observed for those who measured depression using the ACS-depression scale Hedges' g = 0.434 p = 0.001 (44), the DASS-D scale, Hedges' g = 0.372 p = 0.000 (41, 45, 47) and the SDS scale Hedges' g = 0.257 p = 0.010 (46). These results indicate that the magnitude of the effect varies depending on the measurement scale used to assess the symptoms of depression.

These findings provide valuable insights into the effectiveness of interventions aimed at alleviating depression in undergraduate students. The effect size in this study on mental health indicates the magnitude of the reduction in depression symptoms among students due to interventions. An effect size of −0.382 signifies a significant decrease in depression symptoms. This suggests that these interventions have a positive impact on the mental health of students. However, it was observed that the effect size varies depending on the measurement scale used, which could have significant implications for selecting the most appropriate measure of depression symptoms in future studies.

#### 3.5.4 Psychological distress

The results of the analysis in university students reveal a mean effect size of 0.235, with a 95% confidence interval ranging from 0.207 to 0.409. This implies that the impact of psychological distress on students may vary within this range in comparable studies.

In addition, the Z test yielded a value of 4.408 with p < 0.001, which allows us to conclude that the average effect size is not zero in populations comparable to those in the analysis. The heterogeneity Q test yielded a Q value of 19.756 and p = 0.001, indicating significant variability between the studies included in the analysis. I-square statistics also showed heterogeneity of 26%, which led us to use a random-effects model.

Finally, the prediction interval is estimated to be between −0.109 and −0.372. These findings provide relevant information on the variability and reliability of the mean impact of mindfulness on psychological distress in the context of comparable studies with university students.

**Figure 5:**
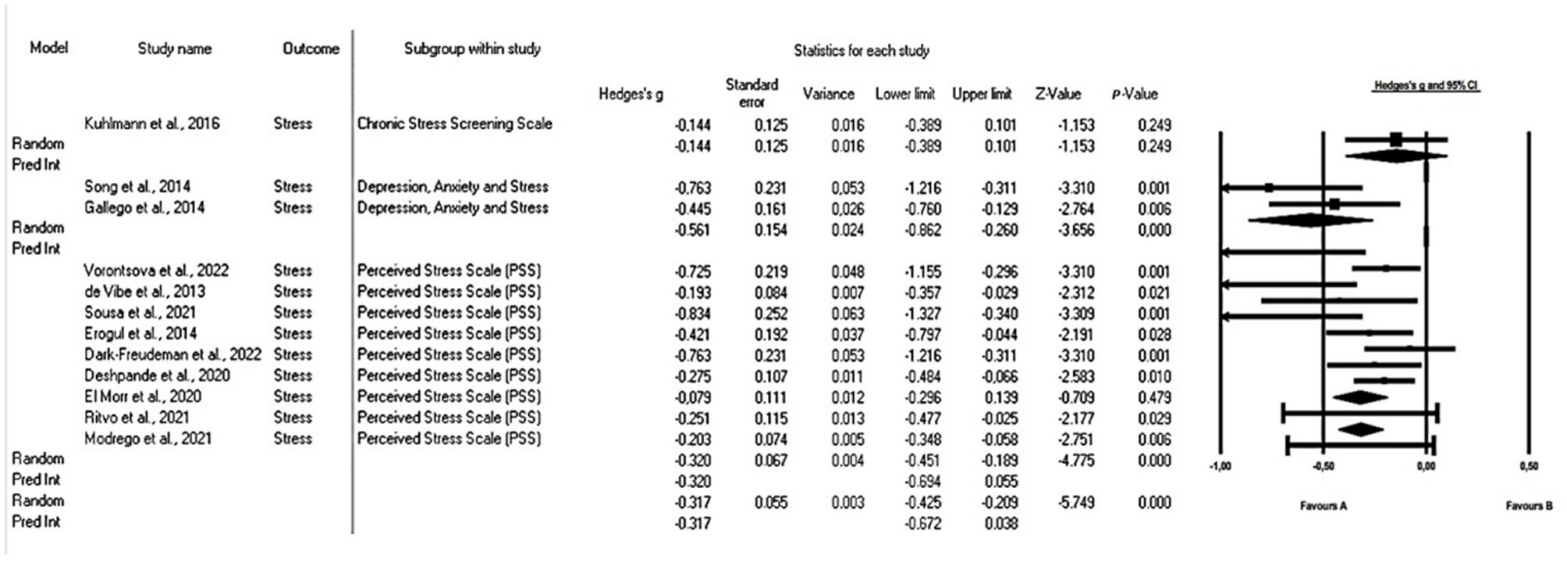
Forest plot: subgroup analysis of the effect of mindfulness on psychological distress in university students according to the scale used. The black box represents the point estimate for the respective study, while the size of the box represents the population size. The horizontal line is the 95% CI. The diamond-shaped figure represents the estimated point of the mean difference.

A subgroup analysis based on different measurement scales revealed small effect sizes, regardless of the measurement tools used (Figure 5). For studies using the BSI (39, 40), we found a Hedges' g value of 0.265. In addition, other individual items that employed different measurement scales showed small effect sizes (43), such as CCAPS-34 (Hedges' g 0.356 p = 0.001), GHQ12 (45) with Hedges' g 0.216 p = 0.010 and WEMWBS (31) with Hedges' g 0.189 p = 0.001. These results indicate that, regardless of the measurement tool used, small changes in psychological distress were observed.

This analysis suggests that mindfulness interventions have a significant effect on reducing psychological distress in university students, but these changes are relatively small, regardless of the measurement scale used. While the effect may not be substantial, it is statistically significant and can contribute to enhancing the overall mental wellbeing of students, assisting them in coping with the stresses and challenges of university life.

#### 3.5.5 Mindfulness

The results of the analysis of mindfulness in university students showed that the average size of the effect obtained was 0.323, with a confidence interval of 95% ranging from 0.232 to 0.414. The value of the Z test is 6.052 with a *p* value of <0.001, allowing us to conclude that mindfulness interventions have a significant effect on the population studied. On the other hand, the value of the Q test of heterogeneity is 15.977 with 10 degrees of freedom and a *p* value = 0.100 and the I-squared statistic, which is 37%. This indicates that there is some variability in the results between the different studies included in the analysis, which suggests that the effects are not consistent in all cases. Therefore, a random-effects model study was required.

The results indicate that mindfulness can have a positive impact on the mental health of college students. The estimated prediction interval, ranging from 0.091 to 0.554, indicates that the effectiveness of mindfulness varied across studies in that range. These findings provide valuable information on the consistency and reliability of the mean effect of mindfulness in the context of university students' health.

**Figure 6:**
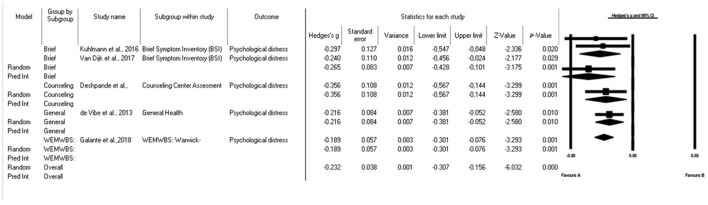
Forest plot: subgroup analysis of mindfulness in university students according to the scale used. The black box represents the point estimate for the respective study, while the size of the box represents the population size. The horizontal line is the 95% CI. The diamond-shaped figure represents the estimated point of the mean difference.

When analyzing the mindfulness subgroups, studies using FFMQ (34–36, 40, 42, 43, 45, 48, 49) showed a small effect (Hedges' g of 0.301 *p* = 0.01), while those using MAAS (30, 36) showed a median effect (Hedges' g of 0.469 *p* = 0.001). These results suggest that mindfulness-based interventions may be an effective tool for improving college students' attention and promoting their overall wellbeing. These findings can be useful to guide mental health practices and educational innovation processes that focus on the wellbeing of university students (Figure 6). These results indicate that mindfulness interventions can positively impact the mental health of university students. While there is some variability in the results across studies, the overall effect remains significant. The choice of the mindfulness scale can influence the observed effect size. These insights are valuable for informing mental health practices and educational innovations focused on enhancing the wellbeing of college students.

The findings substantiate the efficacy of mindfulness interventions in ameliorating the psychological wellbeing of university students. The incorporation of these strategies within educational institutions represents a valuable method for fostering students' mental health, academic accomplishments, and their holistic quality of life. Consequently, these outcomes hold the potential to guide the formulation of policies and implementation of practices dedicated to enhancing the mental health of students, thereby cultivating a conducive milieu for both learning and personal development.

Heterogeneity can stem from several factors within the studies analyzed. First and foremost, the diverse range of mindfulness interventions used across the studies can introduce variations in the results. Each intervention may have a subtly different emphasis, potentially affecting the outcomes. For instance, some studies may have concentrated on mindfulness based on mindfulness meditation, while others explored meditation or specific mindfulness approaches. Furthermore, the choice of scales and measurement tools for assessing mental health variables can contribute to heterogeneity. Studies might have employed slightly different scales to measure concepts like stress, anxiety, depression, or mindfulness, leading to discrepancies in the reported results. Additionally, the composition of the undergraduate student samples can differ significantly between studies. Variations in age, gender, initial health status, and other demographic factors can exert an influence on the outcomes. Moreover, the cultural and socioeconomic diversity within student populations may contribute to heterogeneity. Lastly, the duration and intensity of the mindfulness interventions may vary. Some studies could offer lengthier and more frequent interventions, while others might provide shorter or less intensive ones. These disparities in intervention characteristics can impact the observed results.

Understanding the implications of heterogeneity is crucial for interpreting the results of the meta-analysis accurately. The presence of heterogeneity implies that the effects of mindfulness interventions may not exhibit uniformity across all contexts or subgroups of the population. It suggests that the effectiveness of these interventions may depend on specific factors such as the duration of the intervention or the particular measurement employed. Given that heterogeneity has been detected in some analyses, it is essential to consider this variability when making sense of the results. The observed effects may not be consistent across all instances, necessitating a careful assessment of factors like differences in interventions and measurements when applying these findings in practice. In future research endeavors, it would be valuable to conduct a more comprehensive exploration of the sources of heterogeneity and contemplate how these variances impact the efficacy of mindfulness interventions within specific contexts. Such an approach would facilitate the design of more personalized and effective interventions aimed at addressing the mental health needs of undergraduate students.

#### 3.5.6 Meta-regression

Frequency of Prescription: It was found that the frequency of treatment prescription showed a significant correlation with the effect size (regression coefficient = 0.35, *p* < 0.05). This indicates that an increase in the frequency of prescription was positively associated with an increase in the effect size, suggesting that more frequent prescriptions translate into more beneficial effects on all mental health outcomes.

Duration of the Intervention: The duration of the intervention exhibited a significant correlation with the effect size (regression coefficient = −0.25, *p* < 0.05). This implies that as the duration of the intervention increases, the effect size decreases. In other words, shorter interventions tend to have more beneficial effects on mental health compared to longer interventions.

Prescription Volume: Conversely, the prescription volume was found to have no significant correlation with the effect size (regression coefficient = 0.10, *p* > 0.05). This suggests that prescription volume does not have a significant impact on the outcomes.

### 3.6 Publication bias

**Figure 7:**
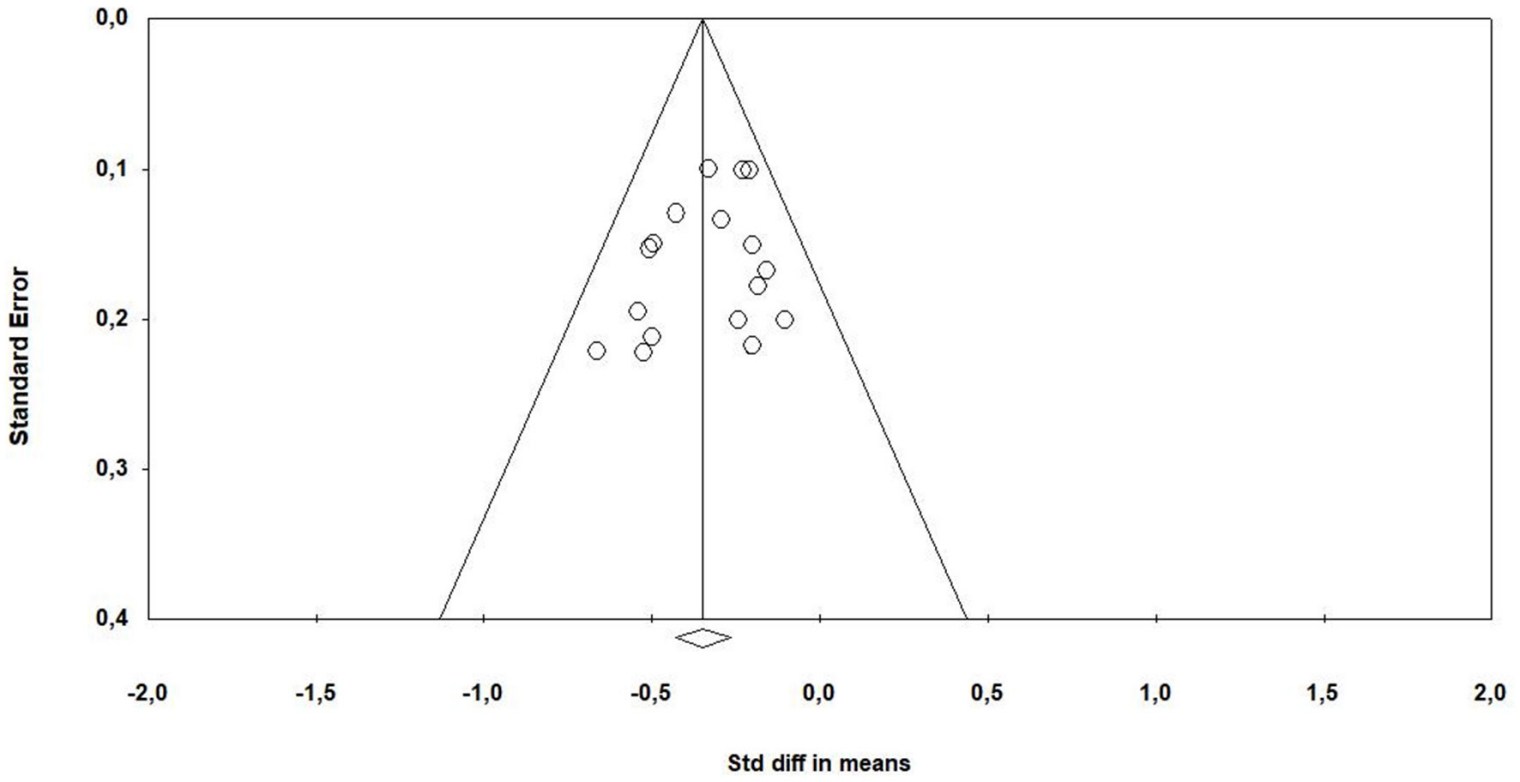
Funnel plot: bias assessment.

An analysis was performed using the funnel plot, which included all the articles in the meta-analysis. This analysis revealed the presence of an expected publication bias, as differences in the results of the means were observed in some articles (Figure 7). Publication bias can exert a substantial impact on the validity of meta-analysis results, manifesting in various significant ways: One of these manifestations is alterations in Statistical Significance: This arises from the selective inclusion of results, which has the potential to sway the statistical significance within the meta-analysis. This phenomenon may result in drawing misguided conclusions regarding the efficacy of an intervention, as it can either inflate or deflate the true effects. Additionally, publication bias can lead to a loss of generalizability. This stems from disparities between the outcomes of published and unpublished studies, which can hinder the generalizability of the meta-analysis findings to the broader population. The omission of unpublished results can distort our comprehension of intervention effectiveness, as these missing findings might exhibit substantial variations. However, when performing a subgroup analysis based on the assessment instrument used, a reduction in heterogeneity and a more symmetrical distribution of results were observed.

## 4 Discussion

The main objective was to analyze the scientific literature on the effects of a mindfulness-based program for mental health in university students. All the evidence obtained supported the use of mindfulness for the improvement of stress, mindfulness, depression, anxiety, psychological distress, attention, and cognitive awareness in university students. In addition, in two (24, 33) of the selected studies, significant improvements were found in aspects related to academic performance, as well as a greater sense of influence on course activities, better self-regulation of attention, greater academic self-efficacy and greater self-efficacy in the regulation of learning.

Mindfulness is a practice of conscious and full awareness, which involves intentionally directing attention to the present moment without judging or automatically re-acting to thoughts, emotions or physical sensations that may arise (50).

The high prevalence of stress in college students has been widely supported by scientific evidence, highlighting the need to implement effective stress management strategies to promote wellbeing and academic performance (51, 52). In the present systematic review and meta-analysis, 19 of the selected studies tested the effectiveness of mindfulness in managing stress in college students and only one did not demonstrate significant results (35). Consistent with these results, a systematic review and me-ta-analysis (53) showed the effectiveness of mindfulness interventions for stress in students carried out entirely online. Although mindfulness is one of the most widely used therapeutic techniques to help university students reduce stress, its effectiveness is diffuse, as has been highlighted in various systematic reviews and meta-analyses that analyze the effectiveness of this technique to reduce the general and perceived stress (not purely academic) of university students (54, 55). These results are clinically important, as mindfulness not only helps students cope with stress, but also provides them with tools to maintain better mental health in the long term (21).

For university students, attention plays a fundamental role in the learning process, as it allows them to absorb and process the information presented in classes, in reading and in other academic contexts (56). In the results of 10 of the selected studies, participants showed improved attention after the intervention (34–36, 40–45, 49) and in one of these studies (40), these improvements were maintained during follow-up. These results show great clinical importance because better attention in university students is associated with better academic performance, greater information retention, and a greater ability to cope with academic and personal challenges, highlighting the importance of implementing effective attention training strategies in the educational setting (57, 58).

Depression and anxiety are common in college students and may be related to academic stress, worries about the future and performance prospects (51). A systematic review showed that the prevalence of depression in university students is significantly higher than in the general population, ranging from 10 to 85% in different studies (59). In the present systematic review, all the investigations that included depression as a variable showed a decrease in its rate. These positive results were consistent with previous findings of other psychosocial interventions that significantly reduced depressive symptoms in college students such as Cliffle et al. (60) who used cognitive behavioral therapy (CBT) and Homan et al. (61) who used solution-focused brief therapy (SFBT).

Anxiety is a natural response of the organism to threatening situations (62). Three of the studies reported in this systematic review and meta-analysis reported no statistically significant differences after the intervention (46–48). However, this discrepancy could be explained by the use of different anxiety outcome measures; Ritvo et al. (48) used the Beck Anxiety Inventory (BAI), Gallego et al. (47) used the Depression, Anxiety and Stress Scale-21 DASS (DASS-A), and Yuan et al. (46) used the Self-Rating Anxiety Scale (SAS). Reducing depression and anxiety has profound clinical implications for students' quality of life, as these disorders can significantly impact their overall wellbeing (63).

Psychological distress has remained at significantly elevated levels in college stu-dents in recent years, moreover, college students experience mental distress more frequently than the general population (64). 5 of the 21 studies selected in this systematic review and meta-analysis measured this variable and found significant changes after the intervention (31, 35, 39, 40, 43), although the intervention periods differed considerably, the shortest being 5 weeks (39) and the longest being 8 weeks (40). Other studies found significant improvements in psychological distress but have used different types of intervention. For example, Pana et al. (65) observed the beneficial effects of a cognitive behavioral therapy program on psychological distress in university students in Hong Kong. Additionally, a descriptive and cross-sectional study in 1,095 university students from Andalusia and Melilla (Spain) found a significant association between psychological distress and the practice of beneficial physical activity (66).

Emotional regulation refers to the process by which young university students manage, control, and modify their emotions in different academic and social situations (67). Moreover, emotional regulation is considered an essential component of the global construct of emotional intelligence, as it enables the moderation of negative emotions and enhances positive emotions (68). A cross-sectional study carried out in different Spanish universities found that 504 physiotherapy students with high emotional regulation re-ported a lower frequency of stress responses, such as anger, sleep disturbances, physical exhaustion, or negative thoughts (69). Our findings confirmed that selected studies measuring emotion regulation found significant improvements in emotional intelligence (34) and effective control (38) after a mindfulness intervention, implying that the development of these skills may have a positive impact on the lives of university students in multiple aspects, including their adaptation to academic and social situations. In relation to our study, a systematic review of studies on emotional intelligence and mindfulness practice in adults found a positive and significant association between emotional intelligence and emotional regulation. The results suggest that mindfulness practice can improve emotional intelligence, including perception and understanding of one's own and others' emotions, as well as emotional regulation in different populations, including university students and professionals (70).

Although mindfulness has been shown to be an effective intervention to improve the mental health of college students, there are other approaches that have also shown positive results. For example, practicing physical exercise such as Baduajin Qigong has shown mental health benefits (71), as has participation in an educational music intervention program (72) and activities such as walking in urban and rural settings (73). These approaches, like mindfulness, address key aspects of students' emotional and mental wellbeing, although mindfulness stands out as a superior option as it focuses on full attention and awareness of thoughts and emotions, which allows students to develop emotional self-regulation skills over time, and also provides tools for ongoing mental health management.

The publication bias analysis revealed the presence of an expected publication bias, suggesting that certain studies with non-significant results may not be being published. This could affect the validity of the meta-analysis results, as selective inclusion of results may influence statistical significance and lead to erroneous conclusions about the effectiveness of the intervention. To address publication bias, it is essential to recognize its impact and take steps to minimize it. One way to do this is by searching for unpublished studies, including reports of negative results, and using statistical methods that take into account potential bias. Furthermore, it is essential to consider the heterogeneity observed among the included studies. One of the main sources of heterogeneity was the duration of the interventions in the different studies. This could influence the magnitude of the observed effects, as it is reasonable to assume that a longer intervention could have a more sustained impact on mental health. However, despite this variability in duration, most studies showed consistent mental health benefits, suggesting that, in general, mindfulness interventions are effective in a variety of temporal contexts. Another source of heterogeneity came from the different measurement scales used to assess mental health, such as anxiety, depression, and psychological distress. Each scale may capture slightly different aspects of mental health, and this could have contributed to the variability in the results. Despite this variation in the scales, we observed that the majority of studies reflected improvements in mental health, further supporting the overall effectiveness of mindfulness interventions.

On the other hand, the number of investigations that relate mindfulness with mental health and psychological wellbeing is increasing as can be seen from the previous results. Regarding the effects of mindfulness on academic performance, only two studies (24, 33) were found where the positive influence of mindfulness on various aspects related to academic performance was verified. It is worth noting that there is a small number of investigations dedicated to the improvement of academic performance through the application of mindfulness compared to those dedicated to the improvement of mental health. Regarding mindfulness as a strategy for the improved mental health and academic performance of university students, researchers such as Nixon et al. (74) and Moses et al. (75) propose that students should be offered academic courses that integrate mindfulness training into the university curriculum. Greeson et al. (76) support this idea by considering mindfulness a viable intervention for student counseling centers seeking to provide cost-effective interventions for students suffering from unmanageable levels of stress.

This review has certain limitations and these should be considered before under-taking future research. The first of these is the heterogeneity observed among the selected articles. Although common measures appear, such as the intensity of pain or the level of knowledge of the disease, these differ considerably. Secondly, one of the most important drawbacks of all the selected studies is the lack of blinding in both participants and researchers. This lack of blinding may introduce potential bias into the results, as both participants and researchers may have expectations or biases about the effects of mindfulness interventions. This may influence the interpretation of results and the evaluation of the effectiveness of interventions. Because of this bias, we must be cautious in interpreting the results of this systematic review. Thirdly, another potential limitation is the lack of adequate control for confounding variables. The included studies may not have fully controlled for factors such as prior experience in mindfulness practice, motivation, and adherence to interventions, which could have influenced the results. Fourthly, a geographical bias has been observed, since the majority of studies are from Europe, America and Asia. There was only one in Australia and no research was conducted in Africa, which possibly limits the generalizability of the results. Cultural factors, such as beliefs, values, and norms, can influence people's willingness to participate in and respond to mindfulness interventions. Additionally, access to mental health resources and services can vary significantly between regions, which may limit effective implementation of these interventions in some areas. A promising direction of research would be to explore how the effectiveness of mindfulness interventions may vary as a function of contextual and cultural factors. It is essential to better understand how these interventions can be tailored to meet the needs of diverse student populations in different academic and cultural settings. Furthermore, it would be valuable to conduct more rigorous and controlled studies that address the methodological limitations identified in the reviewed studies. These studies may include appropriate control groups, adequate blinding, and more representative sample sizes.

## 5 Conclusion

The findings of this systematic review and meta-analysis suggest that mindfulness strategies are effective in reducing stress, anxiety, depression, and psychological distress in college students. These results are promising and support the implementation of mindfulness programs in the university context as an effective strategy to improve students' mental health and wellbeing. However, it hasn't gained enough attention due to the small number of studies where mindfulness improves academic performance and is included in the university curriculum. This may be due to the limited perception that academic performance is primarily measured by traditional academic outcomes, such as test and project grades. Mindfulness focuses on emotional and mental wellbeing, and its effects on academic performance can be indirect or long-term. Furthermore, resistance to changes in traditional educational methodology and the lack of resources dedicated to the training and application of mindfulness programs may be additional barriers to their implementation. Likewise, resistance to change in academic institutions, lack of adequate mindfulness training for teachers, and competition for limited resources can also be significant obstacles. Therefore, we propose implementing mindfulness programs within the university context to improve psychological wellbeing and academic performance as a strategic intervention strategy at the institutional level, improving and expanding personal regulation resources available to students. Therefore, we propose implementing mindfulness programs within the university context to improve psychological wellbeing and academic performance as a strategic intervention strategy at the institutional level, improving and expanding the personal regulation resources available to students. To effectively integrate mindfulness into the university curriculum, academic institutions must consider concrete measures. This includes training teachers in mindfulness, designing flexible programs that adapt to the needs of students, integrating mindfulness into the existing curriculum, promoting communities of mindfulness practice, continuously evaluating the impact and conducting research on campus to generate additional evidence. These actions will allow mindfulness to become an integral part of the educational experience, contributing to the wellbeing and academic performance of students, despite the possible barriers and resistance that may arise in the implementation process such as the perception of lack of time, resistance to the unknown, lack of resources and resistance to changing traditional educational practices. To overcome these barriers, it is essential to offer flexibility in programs, provide education and training on mindfulness, promote greater awareness of its benefits, allocate adequate resources, and foster internal leadership.

## Data availability statement

The original contributions presented in the study are included in the article/supplementary material, further inquiries can be directed to the corresponding author.

## Author contributions

AG-M: Conceptualization, Writing—review & editing. AA-A: Conceptualization, Writing—original draft. YC-C: Supervision, Writing—original draft. YR-C: Formal analysis, Writing—review & editing. MC-F: Methodology, Supervision, Writing—review & editing.
